# Fear of COVID-19 in Patients with Acute Myocardial Infarction

**DOI:** 10.3390/ijerph18189847

**Published:** 2021-09-18

**Authors:** Marco Marotta, Francesca Gorini, Alessandra Parlanti, Kyriazoula Chatzianagnostou, Annamaria Mazzone, Sergio Berti, Cristina Vassalle

**Affiliations:** 1 Fondazione CNR-Regione Toscana G. Monasterio, 54100 Massa, Italy; marco.marotta@ftgm.it (M.M.); alessandra.parlanti@ftgm.it (A.P.); zulachat@ftgm.it (K.C.); annamaria.mazzone@ftgm.it (A.M.); berti@ftgm.it (S.B.); 2 Institute of Clinical Physiology, National Research Council, 56124 Pisa, Italy; fgorini@ifc.cnr.it; 3 Fondazione CNR-Regione Toscana G. Monasterio, Via Moruzzi 1, 56124 Pisa, Italy

**Keywords:** COVID-19, fear, acute myocardial infarction, distress questionnaires

## Abstract

A marked decline in myocardial infarction (AMI) hospitalizations was observed worldwide during the COVID-19 outbreak. The pandemic may have generated fear and adverse psychological consequences in these patients, delaying hospital access. The main objective of the study was to assess COVID fear through the FCV-19S questionnaire (a self-report measure of seven items) in 69 AMI patients (65 ± 11 years, mean ± SD; 59 males). Females presented higher values of each FCV-19S item than males. Older subjects (>57 years, 25th percentile) showed a higher total score with respect to those in the first quartile. The percentage of patients who responded “agree” and “strongly agree” in item 4 (“I am afraid of losing my life because of the coronavirus”) and 3 (“My hands become clammy when I think about the coronavirus”) was significantly greater in the elderly than in younger patients. When cardiovascular (CV) patients were compared to a previously published general Italian population, patients with CV disease exhibited higher values for items 3 and 4. Measures should be put in place to assist vulnerable and high CV risk patients, possibly adding psychologists to the cardiology team.

## 1. Introduction

Attention to the COVID-19 pandemic has strained the health care system globally, making it difficult to care for patients, especially the most vulnerable subgroups, such as people with acute diseases, including acute myocardial infarction (AMI). In particular, during the COVID-19 outbreak, AMI hospitalizations experienced a “drop” compared to pre-pandemic admission rates, a phenomenon observed worldwide [1,2].

The reasons for this finding are not entirely clear. For example, the decrease in air pollution levels due to quarantine measures may have played a role, although a true decrease in acute cardiac events appears very unlikely [1,2]. Instead, greater patient concern about a referral to hospital emergency departments was suggested as a critical reason for the decline in AMI admissions [1,2]. Of note, AMI patients may present a delayed time from the onset of symptoms to the first medical contact due to the fear of a possible in-hospital infection, as found in Italian and Chinese patients by us and by other researchers [2,3]. As a result, these patients may have a more severe infarction and more complications.

During the COVID-19 outbreak, specific psychometric tools were developed and validated to assess COVID-19 fear [4,5]. In particular, the Fear of COVID-19 Scale (FCV-19S), obtained from a seven-item questionnaire (total score between 7 and 35, a higher sum score indicating greater fear of COVID-19), was validated and applied in different general populations (both Asian and European), and showed significant associations of fear with stress, anxiety, and depression, as assessed by specific validated questionnaires (e.g., Hospital Anxiety and Depression Scale, Perceived Vulnerability to Disease Scale) [4,6,7]. As for the Italian population, the FCV-19S was previously validated in a cohort of 249 participants (age 18 to 76 years), showing significant and positive correlations with the Hospital Anxiety and Depression Scale (HADS, r = 0.649) and Severity Measure for Specific Phobia—Adult (SMSP-A, r = 0.703) [8]. A study in a large Chinese general population found an elevated level of stress, anxiety, and depression (8.1%, 28.8%, and 16.5%, respectively) during the COVID-19 epidemic, and it remained unchanged at the outbreak peak, four weeks later [9]. Likewise, approximately 25% of 7,143 Chinese students experienced anxiety during the COVID-19 outbreak [10]. As FCV-19S was administered almost exclusively in general populations, it is interesting to study its results in patients, especially CV individuals, where mood alterations and/or lockdown can worsen lifestyle habits, cause poor adherence to therapy, avoidance of regular checks for stable CVD patients, and delays in hospital access in case of acute events [11]. Accordingly, we previously reported preliminary data obtained through the administration of the FCV-19S questionnaire in CV outpatients during the first pandemic wave, comparing their results with those published relatively to the general Italian population, evidencing higher scores in CV risk patients for both emotional (item 4) and symptomatic fear expression (items 3 and 6) [2,8].The main aim of this study was to evaluate the effect of the COVID pandemic on fear of COVID in patients with AMI through the administration of FCV-19S. Moreover, to identify possible differences between stable and acute patients, FCV-19S scores were evaluated in CV outpatients and in AMI patients.

## 2. Materials and Methods

### 2.1. Population Characteristics

A total of 69 consecutive Italian patients with ST-elevation myocardial infarction (STEMI) (65 ± 11 years, mean ± SD; 59 males) were enrolled at the Ospedale del Cuore G. Pasquinucci—Clinical Cardiology Department (Massa, a city in *Tuscany*, *which* is a region in *central Italy*) in the period November 2020–May 2021, interspersed with more or less rigid lockdown periods. From January 2021, access to vaccinations was possible, first for healthcare personnel, then progressively for other worker categories (e.g., schoolteachers, etc.). In this time period, lockdown included variable and progressive limitations, which often targeted the restricted territory of the region in which infections were higher, differently from the strict nationwide lockdown during the first wave. In addition, 30 CV subjects afferent to the cardiology outpatient clinic in the period November 2020–May 2021 (62 ± 6 years) and 30 CV outpatients (64 ± 8 years) afferent to the cardiology outpatients clinic of the Ospedale del Cuore G. Pasquinucci during the first COVID-19 wave for the regular periodic check were also evaluated [2].

The definition of STEMI follows the published SC/ACCF/AHA/WHF guidelines for STEMI criteria and management [12].

Standard therapy (e.g., aspirin, beta-blockers, angiotensin-converting enzyme inhibitors, diuretics, statins) was administered to all eligible patients.

### 2.2. Criteria of Patient Eligibility

Patients were considered eligible to be enrolled in the study on the basis of the following inclusion criteria: (1) Adult male and female patients, admitted to the coronary care unit (CCU) for chest pain and subsequently ascertained STEMI. Exclusion criteria were as follows: (1) Severe systemic diseases; (2) Systemic inflammatory disease; (3) Patients refusing or unable to supply written informed consent.

### 2.3. Fear of COVID-19 Scale

The FCV-19S is a self-report measure aimed at assessing fear of COVID-19, and the scale is made up of seven items related to emotional (Items 1, 2, 4, 5) and symptomatic (Items 3, 6, 7) fear reactions to the pandemic [4].

Specifically, patients were asked to answer the following items:

1. I am most afraid of coronavirus;
2. It makes me uncomfortable to think about coronavirus;
3. My hands become clammy when I think about coronavirus;
4. I am afraid of losing my life because of coronavirus;
5. When I watch news and stories about coronavirus on social media, I become nervous or anxious;
6. I cannot sleep because I’m worrying about getting coronavirus;
7. My heart races or palpitates when I think about getting coronavirus.

Participants were asked to respond on a five-item Likert-type scale ranging from 1 (strongly disagree) to 5 (strongly agree), with responses including “strongly disagree” (1), “disagree” (2) “neutral” (3), “agree” (4) and “strongly agree” (5). The minimum possible score for each question is 1, and the maximum is 5. The total score ranges between 7 and 35, with a higher sum score indicating greater fear of COVID-19. The measure showed appropriate internal validity (Cronbach’s alpha of 0.82) and was also found to correlate with anxiety and depression, as assessed by the Hospital Anxiety and Depression Scale and the Perceived Vulnerability to Disease Scale. Previous studies [4,5,6,7,8] reported FCV-196ì5 validation in different general population cohorts.

In order to assess anxiety and depression for COVID-19, all enrolled patients completed a COVID fear questionnaire at hospital admission as part of routine clinical practice. The survey took around 10–15 min to complete.

Informed consent was obtained from each patient (or their relatives where necessary), and the study was approved by the local Ethics Committee (number 19214, 11 February 2021).

### 2.4. Statistical Analysis

Data are expressed as the mean ± standard deviation. Statistical analyses included Student’s t-test (to determine the significance of the difference between the means of two data sets, sample size n-1 gives degrees of freedom to estimate variability), χ^2^ test (to verify any significant difference between the expected frequencies and the observed frequencies in one or more categories of a contingency table), and linear regression (to evaluate whether there is a relationship between the variables of interest). A comparison between the three groups for total score and items was tested by using ANOVA analysis and a Scheffe’s post hoc test.

A *p*-value of 0.05 was considered statistically significant. Analyses were performed using Statview statistical software version 5.0.1 procedures (Abacus Concepts, Berkeley, CA, USA).

## 3. Results

### 3.1. Total FCV-19S Score

The COVID fear questionnaire was administered to a total of 69 STEMI patients admitted at the period of the beginning of November 2020–end of May 2021 in the coronary care unit (CCU) of the Ospedale del Cuore G. Pasquinucci—Clinical Cardiology Department (Massa, Italy).

No significant differences were observed in the total score values according to gender (Table 1). On the other hand, there was a significant correlation between age and total FCV-19S score (r = 0.2, *p* ≤0.05) in the overall STEMI population. When the STEMI population was divided by the 25th percentile of age corresponding to 57 years (*n* = 52 older *versus n* = 17 younger patients), the total FCV-19S score resulted in 19 ± 7 and 16 ± 5 in older and younger patients, respectively (*p* = 0.08).

The total FCV-19S score did not significantly differ between the two groups of CV patients taken in the different periods and in STEMI Patients (Table 2).

### 3.2. FCV-19S Items

**Figure 1:**
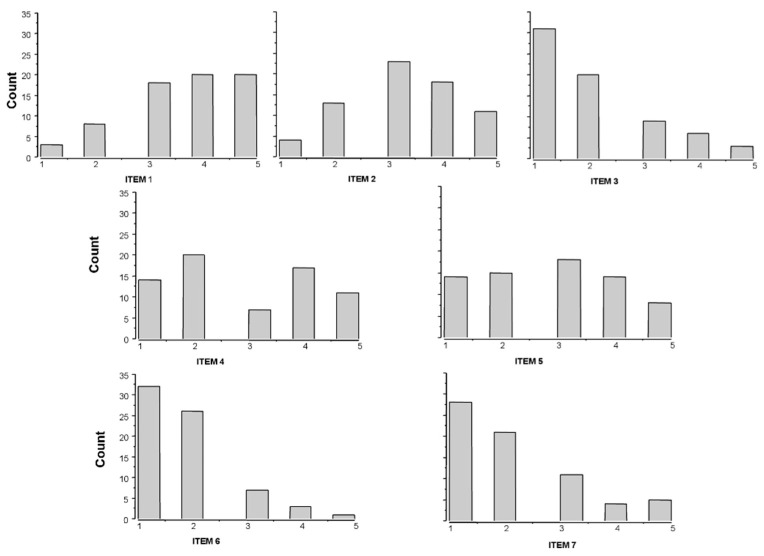
Histograms of the seven items of the Fear of COVID questionnaire in the overall STEMI population.

Histograms of the seven items of the Fear of COVID questionnaire in the overall population are shown in Figure 1. Most of the items were distributed asymmetrically, with the lowest frequencies in the higher value categories.

**Figure 2:**
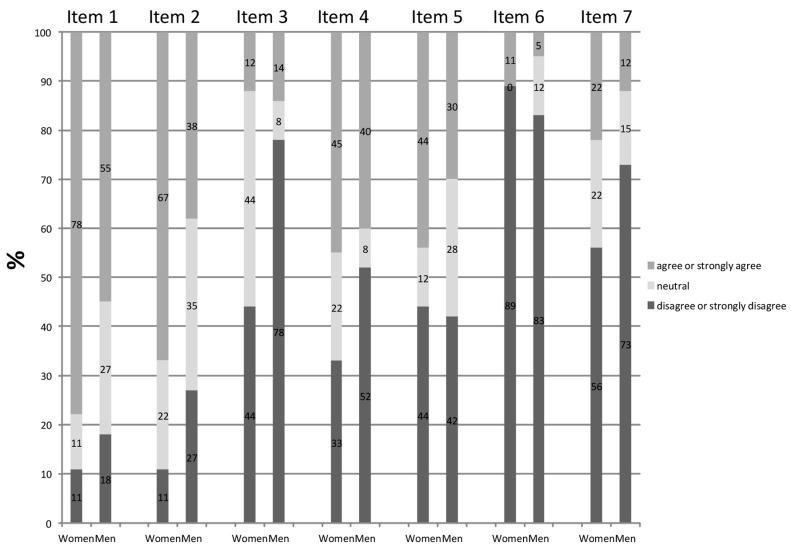
Histograms reporting the % answers corresponding to score 1 and 2 (“strongly disagree” or “disagree”), 3 (“neutral”), or 4 and 5 (“agree” and “strongly agree”) in STEMI female and male patients.

No significant differences were observed when considering each item’s values according to gender (Table 1). The percentage of answers corresponding to score 1 and 2 (“strongly disagree” or “disagree”), 3 (“neutral”), or 4 and 5 (“agree” and “strongly agree”) in both sexes are reported in Figure 2.

**Figure 3:**
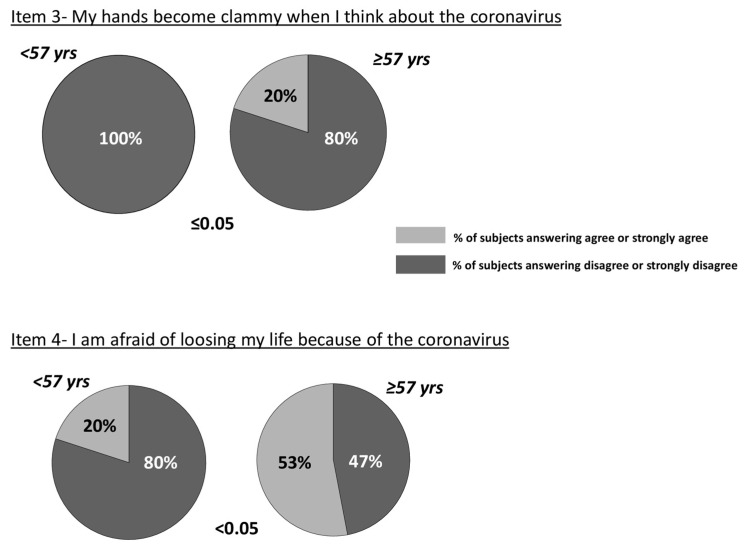
Percentage of STEMI patients answering positively “agree” or “strongly agree” or negatively “disagree” or “strongly disagree” to items 4 and 3 in the groups rated according to the 25th percentile of age (corresponding to 57 years). (Answers reporting “neutral” were excluded by the analysis.) Values are reported as n (%).

There was a significant correlation between age and item 4 (“I am afraid of losing my life because of the coronavirus”) (r = 0.3, *p* < 0.05) in the overall STEMI population. Moreover, when the STEMI population was divided by the 25th percentile of age corresponding to 57 years, the percentage of subjects who responded positively (“agree” or “strongly agree”) compared to those who responded negatively (“disagree” or “strongly disagree”) to item 4 (“I am afraid of losing my life because of the coronavirus”, belonging to emotional fear reactions, *p* < 0.05) and 3 (“My hands become clammy when I think about the coronavirus”, belonging to the symptomatic expression of fear, *p* ≤ 0.05) was significantly higher in the STEMI elderly patient group (Figure 3).

When CV patients referred to the outpatient department during the first period of the COVID-19 outbreak (2) were compared to 30 CV outpatients examined in the period November 2020–May 2021, item 6 (“I cannot sleep because I’m worrying about getting the coronavirus“) and 7 (“My heart races or palpitates when I think about getting the coronavirus“), belonging to the category of symptomatic expression of fear, resulted lower as the pandemic progressed (Table 2). STEMI patients also exhibited lower levels for item 6 than CV outpatients tested during the first wave of COVID-19 (Table 2). Furthermore, when CV patients were compared with published FCV-19S scores from an Italian general population previously tested in the first wave of COVID-19 [11], both CV outpatients and AMI patients subsequently tested showed higher values for both emotional (item 4—“I am afraid of losing my life because of the coronavirus”, corresponding to a value of 2 in the general Italian population) and symptomatic fear expression (item 3—“My hands become clammy when I think about the coronavirus”, corresponding to a value of 1.5 in the general Italian population).

## 4. Discussion

To the best of our knowledge, this is the first report to evaluate FCV-19S in patients with STEMI. This is particularly important, as many data have reported a marked reduction in AMI hospitalizations during the first wave of the worldwide pandemic [2,12,13,14,15]. Common international lockdown measures, contradictory and ambiguous information, and inaccurate communications from the media may have fueled fear of possible in-hospital contagion, which may have contributed to the decline in access to the CCU [2]. Clearly, other determinants may have played a role, such as the healthcare focus on COVID patients and the reduction in resources available for other acute emergencies because all efforts directed towards COVID-19, or the decrease in air pollution with the establishment of lockdown measures and consequently its diminished role as potential trigger of acute coronary artery disease, and others [2]. In any case, it is important to consider that STEMI care is strictly time-dependent; thus, any delay in reaching coronary emergency units can increase morbidity and mortality. In fact, the earlier the diagnosis and treatment, the more effective the STEMI treatment, in terms of infarct size and AMI-related complications.

In this context, these are the first data to estimate psychological distress using FCV-19S in CV patients. The values reported for CV outpatients, both in the first wave and in the following period, and in STEMI patients were higher for item 4 (“I am afraid of losing my life because of the coronavirus”) and item 3 (“My hands become clammy when I think about the coronavirus”), compared to those observed in an Italian general population subjected to FCV-19S during the first pandemic wave (corresponding to the values of 2 and 1.5, respectively), indicating a greater emotional and symptomatic fear expression in all CV patients [8]. Certainly, in these types of studies, it must be considered that patients with acute or stable coronary artery disease may have high underlying rates of anxiety and depression that may influence the FCV-19S if compared to the general population [16,17]. However, based on our results when comparing patients in the different time periods (see Table 2), the differences in the FCV-19S response seem more related to the characteristics of the lockdown periods (e.g., information from the media, level of constraints imposed) than to the type of patients (acute *versus* stable), with adverse repercussions for all patients (e.g., lack of checks for stable CV patients, and delays in hospital admission in case of AMI).

For this reason, the analysis of data related to the so-called “Total ischemic time” (a term coined to indicate the time from the onset of chest pain to the first medical contact, arrival at the hospital, and balloon inflation during primary percutaneous coronary intervention) is essential in the interpretation of the present results. Indeed, the uncertainty in recognizing the severity of the symptoms and in reaching the emergency department introduces a “COVID-19-related delay” in the “Total ischemic time”. This is especially true for the “symptom-onset-to-first-medical-contact time” that was significantly longer during the pandemic period than in the pre-pandemic period, as other researchers and we observed in the CCU [2,3,18]. This finding suggests patients’ reluctance to promptly contact healthcare personnel who may intervene with the first treatment, go to the hospital or even not seek care at all, even though this attitude could have a detrimental impact on their outcomes. Noteworthy, “Door-to-hospital-arrival-time” and “Hospital-arrival-to-insufflation-time” did not vary significantly in the pre-COVID or during the pre-pandemic and pandemic periods in all evaluated clinical settings [2,3,18] suggesting a good functioning of the healthcare system, and also giving a major role to the patient’s fear and reluctance for the reduction in AMI.

Regarding gender, we did not find any significant difference in the level of fear, although female patients presented slightly higher values for each item. This result is absolutely preliminary and limited by the low number of women in our cohort (13%) and certainly needs further deepening. In the literature, other data suggested that higher rates of fear among women can be associated with different emotional distress vulnerabilities depending on gender. Women seem more prone to stress, as well as to an increased risk of developing post-traumatic stress disorders [19]. A 2020 WHO report highlighted that women represent a population with specific concerns, as a significantly higher percentage of women reported being stressed than men during the COVID-19 outbreak, evidencing a greater vulnerability of women to the negative impact of the COVID-19 in terms of mental health and wellbeing [20]. Interestingly, data on FCV-19S in different general ethnic populations (Bangladeshi, British, Brazilian, Taiwanese, Italian, New Zealander, Iranian, Cuban, Pakistani, Japanese, and French) showed that females had a greater fear of COVID-19 than males [21].

In addition, a study specifically designed to evaluate gender differences in fear of COVID-19 suggested greater psychological vulnerability in Cuban women during the pandemic, and that gender significantly predicted COVID-19 fear [22].

Accordingly, in the cardiovascular setting where FCV-19S evaluation had not still been performed, a greater reduction in STEMI admissions was observed comparing women *versus* men (41.2%; *p* = 0.011, and 17.8%; *p* = 0.191, respectively) during the COVID-19 pandemic, which may reflect increased fear in female patients [12].

Since COVID-19 and the highest mortality and complication rate were found in elderly subjects during the outbreak, it is not surprising that older CV patients are more likely to be psychologically affected, as reported in several general populations [23,24,25,26,27]. However, some studies reported lower levels of COVID-19 fear in older subjects than in young to middle-aged adults [21].

Nonetheless, patients with CV disease and comorbidities may feel more vulnerable to death and disability due to COVID-19 than their younger counterparts, likely thinking that the treatments for COVID-19 are somewhat limited and become more fearful of being infected from the virus [28,29].

Notably, “fear” may be a physiological and functional response, which represents a positive reaction towards more adaptive functions aimed at keeping oneself safe from risky situations [30]. However, many of the items in the FCV-19S scale are related to anxiety, a negative emotional state with adverse repercussions [30]. Moreover, loneliness is a strong determinant for all-cause mortality in aged people [31].

It is also noteworthy that CV outpatients examined in the period of November 2020–May 2021 showed significantly lower values for item 6 (“I cannot sleep because I’m worrying about getting the coronavirus”) and 7 (“My heart races or palpitates when I think about getting the coronavirus”) than those tested with FCV-19S in the first wave, indicating some kind of addiction to stressful conditions.

Indeed, as it is known in the field of stress neurobiology, a stress, always of the same nature, which repeatedly manifests over time (homotypic stress), typically leads to the habituation of stress-sensitive systems, including those affecting the hypothalamic–pituitary–adrenal axis, and unlike a heterotypic unpredictable and variable stress [32,33].

In fact, if biological responses give our body the strength to facilitate survival and face immediate danger, long-lasting stress can cause problems, potentially compromising the functions of the whole organism [34,35]. Therefore, homotypic stress addiction can reduce the overall burden. Further studies are warranted to understand whether biological responses to COVID-19 also fit into this context, as well as to clarify whether these biological responses can influence psychosocial behaviors.

### Strengths and Limitations

The main strength of this study is that for the first time, the FCV-19S was administered in CV patients, suggesting that COVID-19 fear may contribute to the delay in regular checks and hospital admissions for stable and acute CV patients.

Due to the limited number of participants, it was difficult to conduct subgroup analyses. For example, the gender analysis included only a small number of female patients. However, this fact is representative of the clinical AMI reality, where there is a male:female event ratio of 5:1 [36]. However, despite the lower incidence of acute coronary artery disease in females, women have worse short- and long-term outcomes than men [37,38]. Moreover, the pre-hospital delay from symptom onset to admission is generally significantly longer for women also ordinarily [39].

These aspects, in addition to an overall greater fear of COVID-19 for women compared to males [40], may result in a further delay in hospital access in the case of AMI for women due to the COVID-19 fear, which could worsen their outcomes. Our preliminary results are in agreement with studies conducted in cohorts of general subjects in different parts of the world [21], suggesting that the female gender may represent a critical predictor for psychological distress. Therefore, although limited in sample size, this study can broaden the knowledge and improve understanding of the factors associated with short-term outcomes after AMI hospitalization by being, to the best of our knowledge, the first to assess fear of COVID-19 in AMI patients.

Unfortunately, we did not enroll AMI patients during the first pandemic wave. Nonetheless, in light of the data collected in the period November 2020–May 2020, the differences observed in the items might be attributable more to the characteristics of the lockdown (e.g., more rigorous lockdown measures) than to the differences between stable and acute CV disease.

Of note, all patients belong to Italian nationality, whereas it was reported that migrants and other similar groups showed a particular fear of COVID and may represent an interesting cohort to study also in the CV setting [41].

## 5. Conclusions

The COVID-19 pandemic has an impact not only on the rate but also on the timing of AMI hospital admissions. Since the symptom-onset-to-first-medical-contact time plays a crucial role in a longer delay, and patients presented with higher levels of emotional and symptomatic fear expressions than the general population, a major cause of this delayed presentation could be attributable to changes in patient behavior and risk perception, which arouses reluctance to come to the hospital for fear of contracting COVID-19, as confirmed by the patients themselves in previously published reports [42,43]. Interestingly, recent data have not confirmed the association between a decrease in hospital admissions for acute coronary syndrome and a decrease in air pollution due to lockdown containment measures, indirectly giving strength to other hypotheses for the drop observed in AMI procedures [44].

While it is true that patients may develop some sort of addiction to the fear of COVID-19, measures should be put in place to assist high CV risk and more vulnerable patients and (e.g., women, elderly, frail subjects), along with correct information to patients on the pandemic course and on the risks of delayed access to the hospital in case of acute events. In addition, a multidisciplinary team should be implemented when possible, including not only cardiologists and hemodynamics but also psychologists (to provide psychological support to CV patients and reduce distress and subsequent mental problems), in order to avoid patients who have presented too late and are hemodynamically unstable for COVID fear as well as AMI complications.

## Figures and Tables

**Table 1:** Descriptive analysis of the Fear of COVID-19 items by gender.

	Overall Population				Women				Men			
	Mean	Standard Deviation	Skewness	Kurtosis	Mean	Standard Deviation	Skewness	Kurtosis	Mean	Standard Deviation	Skewness	Kurtosis
Item 1	3.7	1.1	−0.5	−0.6	4	1	−0.8	−0.2	3.6	1.2	−0.4	−0.6
Item 2	3.6	1.1	−0.4	−0.6	3.8	0.97	−0.4	−0.6	3.2	1.1	−0.1	−0.7
Item 3	2	1.2	0.5	−0.5	2.4	1.3	0.46	−0.5	1.9	1.1	1.2	0.5
Item 4	2.9	1.4	0.1	−1.4	3.1	1.3	−0.2	−1	2.9	1.4	0.2	−1.4
Item 5	2.8	1.3	0.1	−1.1	3	1.6	0.1	−1	2.8	1.3	0.1	−1
Item 6	1.8	0.9	1.3	1.6	2	1.2	1.7	2.2	1.7	0.9	1	0,3
Item 7	2.1	1.2	1	0.2	2.6	1.6	0.6	−1	2	1	1.1	0.4
Total score	18.5	6.6	0.4	−0.4	20.7	7.4	0.5	−0.4	18.2	6.5	0.4	−0.5

**Table 2:** Mean (± standard deviation) of the 7 items of the Italian Fear of COVID-19 score in CV outpatients during the first epidemic wave and the period of November 2020–May 2021.

	First Wave CV Outpatients	November 2020–May 2021 CV Outpatients	November 2020–May 2021 STEMI Patients
Emotional fear reactions			
1. I am most afraid of the coronavirus	3.5 (1.3)	3.8 (1.3)	3.7 (1.1)
2. It makes me uncomfortable to think about the coronavirus	3.2 (1.6)	3.0 (1.2)	3.3 (1.1)
4. I am afraid of losing my life because of the coronavirus	2.9 (1.6)	2.7 (1.2)	2.9 (1.4)
5. When watching news and stories about the coronavirus on social media, I become nervous or anxious	3.0 (1.8)	2.4 (1.3)	2.8 (1.3)
Symptomatic expression of fear			
3. My hands become clammy when I think about the coronavirus	2.1 (0.7)	1.9 (0.9)	2.0 (1.2)
6. I cannot sleep because I’m worrying about getting the coronavirus	2.2 (0.8)	1.4 (0.3) **	1.8 (0.9) *
7. My heart rates or palpitates when I think about getting the coronavirus	2.4 (1.0)	1.6 (0.4) *	2.1 (1.2)
Total mean	2.8 (1.0)	2.4 (0.7)	2.6 (1.0)
Total score	19.5 (6.7)	16.7 (5.1)	18.5 (6.6)

* *p* < 0.1, ** *p* < 0.05 vs. first wave CV outpatients.

## Data Availability

Data available on request from the authors.
